# Intravoxel Incoherent Motion applied to Cardiac diffusion weighted MRI using breath-hold acquisitions in healthy volunteers

**DOI:** 10.1186/1532-429X-14-S1-P261

**Published:** 2012-02-01

**Authors:** Benedicte M Delattre, Magalie Viallon, Hongjiang Wei, Yuemin Zhu, Vinay M Pai, Han Wen, Pierre Croisille

**Affiliations:** 1 CREATIS, CNRS (UMR5220), INSERM (U1044), Université de Lyon, Lyon, France; 2 Department of Radiology, University Hospital of Geneva, Geneva, Switzerland; 3 Department of Radiology, Université Jean-Monnet, Saint-Etienne, France; 4 Imaging Physics Lab, BBC/NHLBI/NIH, Bethesda, MD, USA

## Background

Diffusion weighted imaging (DWI) gives rise to a unique method to evaluate perfusion and diffusion parameters of a tissue without the need of any contrast agent, with the introduction of the Intravoxel Incoherent Motion (IVIM) model (Le Bihan, Radiology 1988). Despite its relevance, cardiac DWI has so far been limited to low b-values primarily due to signal loss induced by physiological motion. Recently, an efficient cardiac DWI method was proposed where images were acquired at different time points of the cardiac cycle and where motion-induced signal-loss was removed by Principal Component Analysis (PCA) filtering and temporal MIP (tMIP) techniques (PCATMIP) (Rapacchi, Invest Radiol 2011). We compared the IVIM parameters obtained at a single optimized diastolic time point of the cardiac cycle (1TD) to the results obtained with PCATMIP technique.

## Methods

Breath-hold DWI scans were performed on 12 volunteers for 10 trigger-delay values in diastole. 13 b-values ranged from 0 to 550 s/mm^2^ were used. Signal intensity (SI) of the LV myocardium was fitted with the IVIM model corrected for T1/T2 relaxation (Lemke, MRM 2010).

## Results

**Figure 1:**
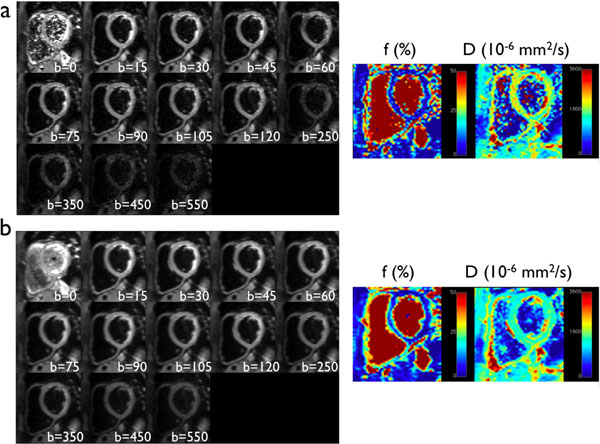
DWIs for 1TD (a) and for PCATMIP (b) with corresponding maps of parameter f and D.

**Figure 2:**
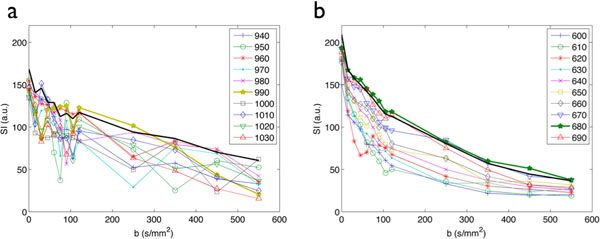
SI for images acquired at 10 different time points (legend indicates the trigger-delay in ms) for (a) volunteer with signal losses due to high R-R variability, (b) volunteer with very stable R-R cycle. Bold lines with star symbols correspond to the trigger delay giving the highest SI (1TD) while the black bold line (without symbols) corresponds to the SI obtained after PCATMIP.

Figure 1 shows examples of DWI for 1TD and PCATMIP as well as maps of IVIM parameters. PCATMIP allowed the recovery of signal loss due to either intra-scan cardiac motion or RR variability over multiple breath-holds (see fig. 2). Perfusion fraction f, diffusion coefficient D and pseudo-diffusion coefficient D* were evaluated. The values of D measured for 1TD and for PCATMIP were similar (2.35±1.12x10^-3^ mm^2^/s and 2.35±0.89x10^-3^ mm^2^/s respectively, p=0.999). However, f was lower with 1TD than with PCATMIP (0.118±0.067 and 0.163±0.056 respectively, p=0.0018). Average D* obtained was 0.106 mm^2^/s for 1TD and 0.0763 mm^2^/s for PCATMIP.

## Conclusions

This study demonstrates feasibility and reports for the first time cardiac IVIM parameters in normal humans. PCATMIP minimized the motion-induced signal loss which is the main problem in cardiac DWI. This study opens new perspectives for perfusion imaging without contrast media.

## Funding

This work was supported by the French National Agency for Research (ANR).

